# *In Vivo* siRNA Delivery and Rebound of Renal* LRP2* in Mice

**DOI:** 10.1155/2017/4070793

**Published:** 2017-12-20

**Authors:** Michael T. Eadon, Ying-Hua Cheng, Takashi Hato, Eric A. Benson, Joseph Ipe, Kimberly S. Collins, Thomas De Luca, Tarek M. El-Achkar, Robert L. Bacallao, Todd C. Skaar, Pierre C. Dagher

**Affiliations:** Department of Medicine, Indiana University School of Medicine, Indianapolis, IN 46202, USA

## Abstract

siRNA stabilized for* in vivo* applications is filtered and reabsorbed in the renal proximal tubule (PT), reducing mRNA expression transiently. Prior siRNA efforts have successfully prevented upregulation of mRNA in response to injury. We proposed reducing constitutive gene and protein expression of* LRP2* (megalin) in order to understand its molecular regulation in mice. Using siRNA targeting mouse* LRP2* (si*LRP2*), reduction of* LRP2* mRNA expression was compared to scrambled siRNA (siSCR) in mouse PT cells. Mice received si*LRP2* administration optimized for dose, administration site, carrier solution, administration frequency, and administration duration. Kidney cortex was collected upon sacrifice. Renal gene and protein expression were compared by qRT-PCR, immunoblot, and immunohistochemistry (IHC). Compared to siSCR, si*LRP2* reduced mRNA expression in PT cells to 16.6% ± 0.6%. In mouse kidney cortex, si*LRP2* reduced mRNA expression to 74.8 ± 6.3% 3 h and 70.1 ± 6.3% 6 h after administration. mRNA expression rebounded at 12 h (160.6 ± 11.2%). No megalin renal protein expression reduction was observed by immunoblot or IHC, even after serial twice daily dosing for 3.5 days. Megalin is a constitutively expressed protein. Although* LRP2* renal mRNA expression reduction was achieved, siRNA remains a costly and inefficient intervention to reduce* in vivo* megalin protein expression.

## 1. Introduction

RNA interference (RNAi) is an attractive approach to transiently cleave mRNA transcripts and ultimately reduce protein expression [1, 2]. For a variety of therapeutic indications, synthetic small interfering RNAs (siRNAs) have been modified to effectively mediate* in vivo* gene and resultant protein expression regulation [3–8]. In the kidney, siRNA stabilized with 2′O-methylation is filtered and reabsorbed in the renal proximal tubule (PT) following IV administration, reducing relative mRNA expression temporarily in the PT [9]. siRNA directed to candidate genes, including p53, has prevented multiple forms of acute kidney injury (AKI) and renal fibrosis in animal models [10–14].

Investigators have succeeded in reducing relative mRNA expression and mitigating disease phenotypes through either high-dose siRNA administration without a carrier molecule or using lower doses bound to nanoparticles [15–18]. Regardless, a common theme is the prevention of candidate gene upregulation in response to injury or disease. Many of these studies reveal extended reductions in transcript expression; however, these reductions are relative to a scrambled control condition in the setting of gene upregulation. In contrast, we proposed reducing gene and protein expression of a constitutively expressed receptor,* LRP2* (megalin), in order to better understand the molecular regulation of this protein.

Megalin, encoded by* LRP2*, is a 600 kDa cell surface endocytic receptor found in the apical membrane of renal proximal tubule epithelial cells [19]. Megalin complexes with cubilin and is important in the reabsorption of trace elements, thyroglobulin, and carrier-bound vitamins [20]. Megalin has been implicated in the uptake of several nephrotoxins, including folate, cadmium, gentamicin, and colistin [21–24]. Receptor activated protein (RAP) and cilastatin have been shown to block the reabsorption of these toxins through megalin-mediated endocytosis [21, 22]. RNAi of* LRP2* may hold similar therapeutic potential.

Although megalin has an extended half-life [25], other investigators have succeeded in providing serial daily or twice weekly administration of siRNA to reduce renal expression of other genes and proteins [11–13]. We hypothesized that serial administration could similarly reduce megalin expression. After significant optimization, successful knockdown of* LRP2* mRNA expression was achieved in mice. However, a prominent rebound effect in* LRP2* mRNA transcript expression was observed. The rebound effect, coupled with the extended half-life of the protein, precluded attaining a reduction in protein expression. We present the data here in order to facilitate optimization for other investigators. Target selection is important. We conclude that the prevention of upregulated expression holds greater feasibility than knockdown of a constitutively expressed protein.

## 2. Methods

### 2.1. Cell Culture

Mouse S1 proximal tubular (mS1PT) cells were previously isolated from a mouse carrying the SV40 large T-antigen transgene [26, 27]. BUMPT mouse proximal tubular cells were a gift from the Patrick Cunningham Laboratory. Cells were maintained in DMEM/F12 (S1) medium (Corning Cellgro, Corning, NY, USA) supplemented with 10% fetal bovine serum (HyClone) and 1% penicillin-streptomycin (MP Biomedicals, Santa Ana, CA, USA). Cells were split when they reached 70–80% confluence and diluted to 20–30% confluency thrice weekly. Mouse proximal tubular cells were maintained at 37°C in 95% humidified atmosphere with 5% CO_2_.

### 2.2. siRNA Nucleofection

mS1PT cells were diluted to 500,000 cells/ml one day prior to nucleofection. Cells were nucleofected using the SF Cell Line Amaxa X-system Nucleofector Kit (Lonza, Inc., Basel, Switzerland) and the CA-137 program on Lonza's 4D-Nucleofector Amaxa X-system. Cells were then centrifuged at 90 g for 10 min at room temperature and resuspended at a concentration of 1,000,000 cells/20 *μ*l in SF/supplement solution (SF Kit, Lonza Catalog V4SC2096) and 2000 nM final total siRNA concentration for one of seven conditions. The seven conditions included (1) All Stars Negative Control siRNA (Qiagen, Inc., Valencia, CA, USA) at 2000 nM concentration, (2) a pool of four Dharmacon si*LRP2* constructs at a 500 nM concentration for each siRNA molecule, (3) through (6) each of the four individual Dharmacon si*LRP2* constructs at a 2000 nM concentration (GE Dharmacon, Lafayette, CO, USA), or (7) an Ambion* in vivo* si*LRP2* molecule at a 2000 nM concentration (Thermo Fisher Scientific, Waltham, MA, USA). Cells were allowed to rest for 10 min prior to the addition of prewarmed (37°C water bath) S1 media and then for another 5 min in the warm S1 media. Cells were then plated for mRNA harvest or drug treatment. mS1PT cells were harvested 24, 48, 72, 96, 120, and 144 h after nucleofection for mRNA expression measurements.

### 2.3. Animals and siRNA Administration

Sixty-nine male C57BL/6NHsd and eight Swiss Webster mice were obtained and studied at 6–8 wk of age (Envigo Laboratories, Indianapolis, IN, USA). All experimentation was approved by the Institutional Animal Care and Use Committee (IACUC) at Indiana University School of Medicine.

Optimization of administration site and carrier solution was assessed. All animals received injections of Ambion* in vivo* si*LRP2*, molecular grade phosphate-buffered saline (PBS) vehicle, Invivofectamine (IVFM) vehicle (IVF3005, Thermo Scientific, Waltham, MA, USA), or scrambled siRNA (On-Target Plus Control Nontargeting siRNA #2 D-001810-02-20, Thermo Scientific). Administrations were provided through tail vein injections, direct intrajugular (IJ) vein injections, or IJ intravenous catheter infusions. All animal studies were conducted with* in vivo* si*LRP2* and animals received doses of either 7.5 mcg/gm or 15 mcg/gm body weight. These doses corresponded to concentrations of 0.75 mcg/*μ*l or 1.5 mcg/*μ*l si*LRP2* prepared in either Invivofectamine or PBS according to the manufacturer's instructions. The initial starting dose of 7.5 mcg/gm body weight was selected to match Ambion's recommended starting dose of* in vivo* siRNA. This dose was also compared to previously published studies of siRNA delivery in mice which were effective at achieving gene knockdown [10–14]. C57BL/6 mice (20 g weight) were used for all experiments except the 3.5-day serial siRNA administration, in which the larger Swiss Webster mice (35 g weight) were employed to tolerate larger daily volumes of siRNA or vehicle. Mice were euthanized by exsanguination and cervical dislocation at appropriate time points with collection of blood, kidney tissue, liver tissue, and lung tissue. Three mice died prior to experimental endpoints due to surgical mortality or because they required euthanasia for catheter-related malfunctions.

### 2.4. Jugular Venous Catheter Placement

Mice were anesthetized with isoflurane. Hair was removed from the surgical site by clippers followed by the hair remover lotion Nair (CVS Pharmacy, Woonsocket, RI, USA) and then disinfected three times with alternating scrubs of iodine and 70% alcohol. A 5–8 mm longitudinal incision was made on the ventral surface of the neck, 5 mm right of the midline. The right jugular vein was exposed and then catheterized by a polyurethane mouse jugular vein catheter (ID, 0.43 mm; OD, 0.69 mm with a collar of 0.9 mm OD, from Instech Laboratories, Inc., Plymouth Meeting, PA, USA) preinfused with PBS. The catheter was secured in place by suturing around the cannulated jugular vein and surrounding tissue, and its free end was connected to a 25 ga PinPort (Instech Laboratories). The catheter and PinPort were tunneled subcutaneously around the neck and exteriorized from the dorsal side. The exposed PinPort was secured on the skin with two sutures and 3M Vetbond surgical glue (Revival Animal Health, Orange City, IA, USA). After surgery, the catheter was flushed with PBS twice daily.

### 2.5. Tissue Collection, Fixation, and Freezing

Kidneys were fixed or frozen for routine histologic analysis, immunohistochemistry, immunoblot, and real-time PCR. Kidneys were obtained at sacrifice, cut transversely, and fixed in formalin or the cortex was isolated and flash-frozen in Eppendorf tubes with liquid nitrogen. The frozen kidney cortex was stored at −80°C for subsequent quantitative real-time polymerase chain reaction (qRT-PCR) and immunoblot analysis. For formalin-fixed tissue, specimens were stored in 4% phosphate-buffered formalin for 6 hours prior to transfer to 70% ethanol. Specimens were then brought to the core pathology lab for embedding, cutting, and immunohistochemistry or periodic acid-Schiff (PAS) staining.

### 2.6. Quantitative Real-Time PCR

qRT-PCR was performed to measure the levels of expression of* LRP2 *in mS1PT cells or in mouse kidney cortex. For cell experiments, a total of 1 million cells were pelleted 24, 48, 72, 96, 120, and 144 h after nucleofection, washed in ice-cold PBS, and centrifuged to remove PBS. All pellets were flash-frozen and stored at −80°C until RNA isolation. Total RNA was extracted using the miRNeasy Plus Mini Kit (Qiagen) following the manufacturer's protocol. RNA was isolated and purified from frozen mouse kidney tissue as previously described [28].

RNA quality assessment and quantification were conducted using the optical spectrometry 260/280 and 260/230 nm ratios. Subsequently, mRNA was reverse transcribed to cDNA using the Bio-Rad iScript Reverse Transcription Kit (Bio-Rad, Hercules, CA, USA). The final concentration of cDNA was 25 ng/*μ*l. qRT-PCR was performed for* LRP2* and* 18S* (used as an endogenous control) with custom made primers (Life Technologies, Carlsbad, CA, USA) and iTaq Universal SYBR Green (Bio-Rad) on the Applied Biosystems ViiA 7 RT-PCR system. Primer sequences are provided in Table 1. The entire reaction was performed in 20 *μ*l volume, which consisted of 10 *μ*l SYBR green, 4 *μ*l cDNA, 0.4 *μ*l of each primer (10 *μ*M stock), and 5.2 *μ*l of water. The thermocycler parameters were 95°C for 30 s, 40 cycles of 95°C for 15 s, and then a lower temperature for 30 s (*18S*: 60°C,* LRP2*: 58°C), with ramping speeds of 1.6–1.98 C/s and a melt curve. The CT threshold and baseline for each experiment were set automatically by the ViiA 7 software.

The delta-delta (ΔΔCT) method was used to obtain the relative expression of each gene. Each sample's expression of* LRP2*  was first subtracted from its* 18S* expression to determine its ΔCT. The ΔCT_scramble_ for cell experiments or the ΔCT_control_ for live animals was then subtracted from the ΔCT_siRNA_ to determine the ΔΔCT. Fold change of the siRNA knockdown as compared to the control was determined by the formula fold change = 2^ΔΔCT^. mRNA expression for each condition is given as a percentage of expression relative to the control condition.

### 2.7. Immunoblot

To assess megalin protein expression, proteins were extracted from either an mS1PT cell pellet or a portion of frozen kidney cortex and stored at −80°C. Pierce Radioimmunoprecipitation assay buffer (Pierce Biotechnology, Rockford, IL, USA) with 1% Pierce protease inhibitor (Pierce Biotechnology, Rockford, lL, USA) was used to prepare protein samples. An immunoblot was performed with 20 *μ*g of protein per lane (*n *= 4 per group) on a NuPAGE® 3–8% Tris-Acetate Gel (1.0-mm, 10 wells, Invitrogen, Life Technologies, Carlsbad, CA, USA). A goat polyclonal antibody to megalin (1 : 500, SC-16478, Santa Cruz Biotechnology, Inc., Dallas, TX, USA) was incubated for 2 hours at room temperature and a secondary donkey anti-goat antibody (1 : 5000, SC-2020, Santa Cruz Biotechnology, Inc.) for 45 minutes. As previously described [29], relative megalin expression was calculated as compared to *β*-actin control (1 : 250, SC-47778, Santa Cruz Biotechnology, Inc.) with ImageJ software (v1.44p, NIH) [30].

### 2.8. Immunohistochemistry

Kidney sections were fixed in 10% neutral buffered formalin (EMD Chemicals Inc., Gibbstown, NJ, USA) for 6 hours at room temperature and then transferred to 70% ethanol. Specimens were paraffin-embedded, sectioned, and stained using the antibody to megalin (1 : 100, SC-16478, Santa Cruz Biotechnology, Inc.), followed by a HRP-conjugated secondary antibody donkey anti-goat (SC-2020, Santa Cruz Biotechnology, Inc.) after deparaffinization and heat antigen retrieval in citrate buffer. Tubular staining intensity was scored by pixel density quantitatively using ImageJ software (minimum* n *= 3 mice, with 10 fields per mouse) and semiquantitatively in terms of intensity on a scale of 0–3 (0 = none, 1 = weakly positive, 2 = positive, and 3 = strongly positive).

### 2.9. Statistics

For cell experiments, statistical significance was assessed based on a Student's *t*-test for two comparisons or ANOVA for 3 or more groups. Between control and si*LRP2* treated animals, a Student's *t*-test was used to compare expression.

## 3. Results

### 3.1. siRNA Targeted to* LRP2* in S1 Renal Tubular Epithelial Cells Reduces Relative mRNA Expression

In order to ultimately achieve* in vivo* knockdown of* LRP2*, the gene encoding megalin, we began with a series of* in vitro* experiments to optimize the siRNA sequence and timing of administration. Baseline* LRP2* mRNA expression was screened in two mouse proximal tubular cell lines, mS1PT and BUMPT, by qRT-PCR. Baseline raw CT values in mS1PT cells ranged from 22.5 to 23.5, while baseline CT values in BUMPT cells ranged from 28 to 30. Due to their significantly higher mRNA expression of* LRP2*, mS1PT cells were selected for downstream experimentation.

**Figure 1:**
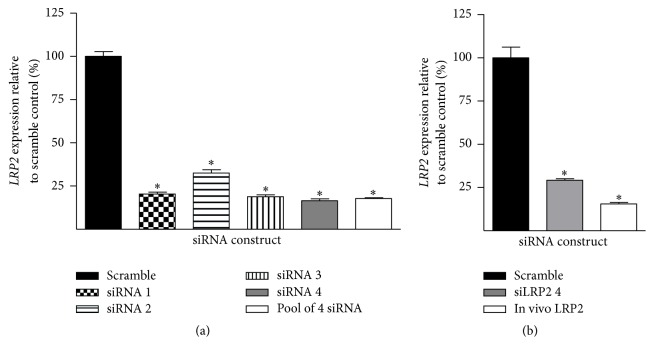
*In vitro* si*LRP2* knockdown leads to reduction of mRNA expression in renal S1 proximal tubule cells. (a) All four commercially available siRNA molecules targeting* LRP2* reduced mRNA expression, individually and as a pool. siRNA #4 led to the strongest knockdown, reducing mRNA expression to 16.6 ± 1.0% (*p* = 4.6 × 10^−10^). (b)* In vivo* si*LRP2* led to significant knockdown in mS1PT cells as well 15.6 ± 0.9%  (*p* = 7.2 × 10^−8^). ^*∗*^*p* < 0.05 as compared to the scrambled control.

Four siRNA sequences targeting* LRP2* were examined individually and as a pool for their efficacy in reducing* LRP2* mRNA expression in mS1PT cells (Figure 1(a)). All sequences significantly reduced mRNA expression as compared to a scrambled control siRNA (siSCR). siRNA sequence 4 led to the most profound reduction in mRNA expression, down to 16.6 ± 1.0% as compared to the scrambled control (*p* = 4.6 × 10^−10^).

The best performing standard si*LRP2* (sequence number 4) was then compared to both the siSCR and a modified Ambion* in vivo* si*LRP2*. Although the Ambion sequence modifications are proprietary, many siRNA constructs created for* in vivo* application utilize 2′O-methylation modifications to stabilize the siRNA against degradation [31]. We examined whether these modifications might impact the efficacy of* in vivo* si*LRP2*. Expression was measured by qRT-PCR (Figure 1(b)) in proximal tubular cells. Both the standard and* in vivo* si*LRP2* molecules significantly reduced mRNA expression as compared to siSCR treatment.* In vivo* si*LRP2* outperformed si*LRP2* sequence 4 in cell culture, eliciting a reduction of* LRP2* mRNA expression to 15.6 ± 0.9% of siSCR treated expression (*p* = 7.2 × 10^−8^).

### 3.2. *In Vivo* si*LRP2* Reduces* LRP2* mRNA Expression for Days in Renal Tubular Epithelial Cells

**Figure 2:**
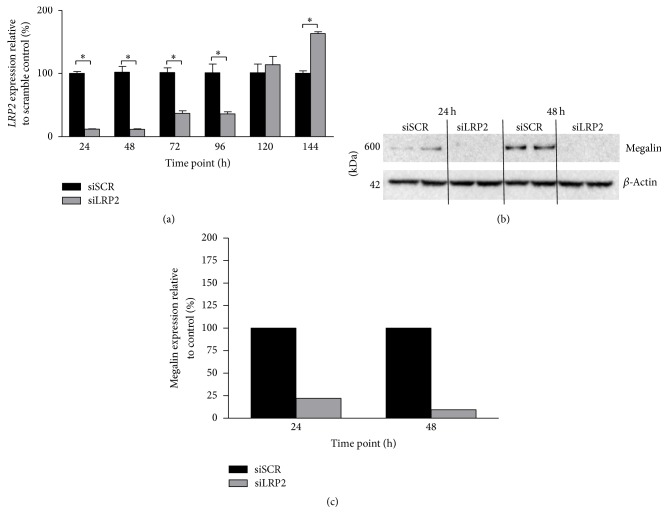
*LRP2* mRNA expression in mS1PT cells is reduced. (a) Knockdown persists for at least 96 h and reaches baseline expression by 120 h (*N* = 3 cell culture experiments). Expression is increased as compared to scrambled (siSCR) expression at 144 h. (b) Megalin immunoblot reveals decreased protein expression in S1 proximal tubular cells treated with si*LRP2*. (c) Expression was reduced to 22.4% 24 h after si*LRP2* administration and 9.1% 48 h after si*LRP2* administration (*N* = 2 cell culture experiments). Densitometry was normalized to a beta-actin control. ^*∗*^*p* < 0.05 as compared to the siSCR control.

We then sought to determine the duration of expression reduction in renal tubular epithelial cells. mS1PT cells treated with* in vivo* si*LRP2* or siSCR were harvested 24, 48, 72, 96, 120, and 144 hours following nucleofection and expression was measured by RT-PCR.* LRP2* mRNA expression remained suppressed for 96 hours following nucleofection (Figure 2(a)). Nadir expression was found 48 h after nucleofection at 11.5 ± 1.0% of scrambled control expression (*p* = 2.0 × 10^−6^).* LRP2* expression was increased in si*LRP2* treated cells as compared to siSCR treated cells at 144 hours after nucleofection (163.3 ± 2.9%, *p* = 1.3 × 10^−7^).

To confirm that knockdown of protein accompanied mRNA expression reductions in proximal tubular cells, protein expression of megalin was measured in untreated mS1PT cells and in cells 24 and 48 hours after nucleofection by immunoblot (Figures 2(b)-2(c)). Abundant megalin expression was found in siSCR mS1PT cells, but not in those exposed to si*LRP2*.

### 3.3. si*LRP2* Reduced Renal mRNA Expression* In Vivo*

**Figure 3:**
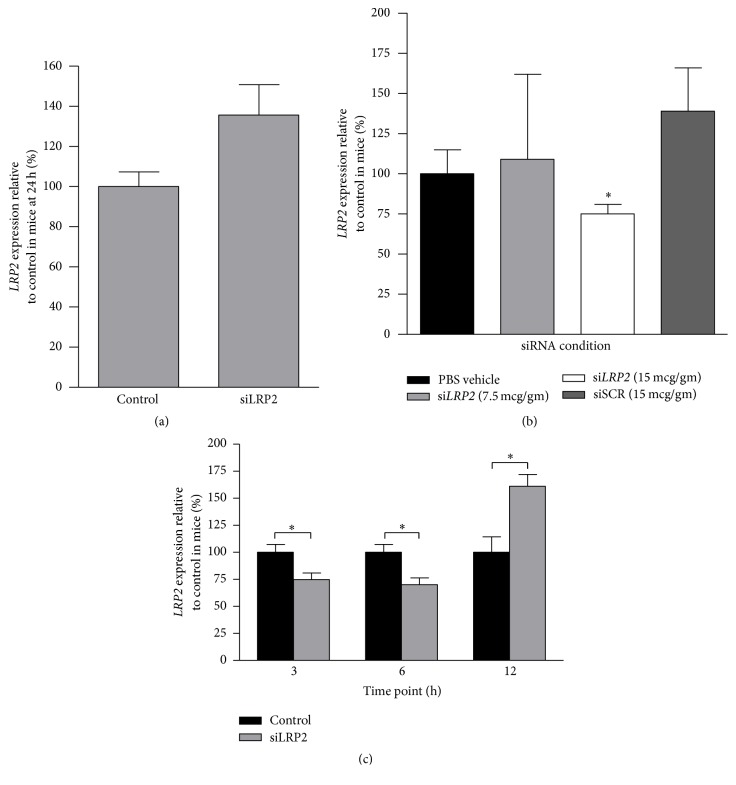
si*LRP2* reduced renal mRNA expression* in vivo*. (a) At 24 h after 7.5 mcg/gm si*LRP2* administration, expression of* LRP2* was not reduced in mouse kidney (*n* = 5 control, *n* = 4 si*LRP2*). (b) At 3 h after siLRP2 administration, mice receiving 7.5 mcg/gm did not have* LRP2* expression reduction. Mice receiving 15 mcg/gm had 25.2% lower* LRP2* expression as compared to control (*p* = 0.034). Mice receiving a modified* in vivo* scrambled siRNA (siSCR) did not have* LRP2* expression reduction (*n* = 4 per group). (c) At 6 h after 15 mcg/gm si*LRP2* administration, mice receiving si*LRP2* had lower expression of* LRP2* as compared to control mice. At 12 h after 15 mcg/gm si*LRP2*, mice had increased expression as compared to control (*n* = 5 control, *n* = 4 si*LRP2* at 6 and 12 h). The 3 h expression data from Figure 3(b) is pictured for reader ease. All siRNA was administered via tail vein and prepared in PBS vehicle. ^*∗*^*p* < 0.05 as compared to the control or PBS vehicle.

While prolonged* LRP2* mRNA suppression was achieved in renal cell culture, the degree of exposure time and concentration of the siRNA in cell culture constructs are unlikely to be achieved* in vivo*. Prior* in vivo* studies have revealed that fluorescently tagged siRNA targeting P53 remains in contact with the renal proximal tubule for at least 2 hours. The siRNA is absent from the nephron at 24 hours, but renal mRNA expression remains reduced for 24 hours [9]. We used the information obtained from the cell culture experiments above, prior published literature, and the siRNA manufacturer's guidelines to conduct animal experimentation. All experimentation was conducted with* in vivo* siLRP2. In order to assess its efficacy, mice were given a single dose of 7.5 mcg/gm body weight of* in vivo* si*LRP2* or PBS vehicle by tail vein injection and mRNA expression was measured 24 h after administration (Figure 3(a)). The dose corresponded to a 3-fold higher amount as compared to historical doses of siRNA delivered to the kidney in PBS [11–13] and corresponds to the manufacturer's highest recommended dose. mRNA expression in mice receiving si*LRP2* was not significantly different from control mice; however an unexpected trend toward increased* LRP2* expression was observed 24 h after mice received si*LRP2* (expression of 135.7 ± 15.1% compared to control, *p* = 0.084).

We considered that the lack of mRNA expression reduction with si*LRP2* may be secondary to (1) restoration of mRNA expression at 24 h, (2) inadequate dose, or (3) inefficacy of the siRNA in mice. We then tested the effect of dose at an earlier time point. Using tail vein injections, mice received a single dose of one of the following: (1) PBS vehicle, (2) 7.5 mcg/gm* in vivo* si*LRP2*, (3) 15 mcg/gm* in vivo* si*LRP2*, or (4) an siRNA negative control. mRNA expression was measured 3 h after administration. At 3 h,* LRP2* expression was not significantly different between mice receiving PBS vehicle and those receiving 7.5 mcg/gm si*LRP2* (Figure 3(b)). However, mice receiving 15 mcg/gm si*LRP2* had 25.2% lower expression of* LRP2* as compared to control mice (expression reduced to 74.8 ± 6.3%, *p* = 0.034). To confirm expression reduction was specific to si*LRP2* administration, a second set of control mice were given an* in vivo* modified scrambled siRNA (siSCR, nontargeting) as a control. Mice injected with siSCR did not have significant* LRP2* expression reduction. A trend toward increased* LRP2* expression was observed (139 ± 26.9%, *p* = 0.082) in mice receiving the siSCR control. Due to the trend toward increased* LRP2* expression in mice receiving the siSCR, PBS vehicle was used as a control in downstream experimentation in order to be more conservative with comparisons. Analogously, the 15 mcg/gm si*LRP2* dose was used in downstream applications, a dose that is 6-fold higher than what most investigators have used to successfully reduce renal mRNA expression in the past [11–13].

We then examined the duration of effect of the* in vivo* si*LRP2 *(Figure 3(c)). Mice were given a single dose of si*LRP2* at 15 mcg/gm or PBS vehicle and sacrificed at 6 h and 12 h following tail vein administration. At 6 h,* LRP2* expression was reduced to 70.1 ± 6.3% compared to control (*p* = 0.015). At 12 h, LRP2 expression was significantly higher in mice receiving siLRP2 as compared to control (expression of 160.6 ± 11.2% compared to control, *p* = 0.002).

### 3.4. *LRP2* Renal Expression Knockdown Is Not Dependent upon Administration Site

**Figure 4:**
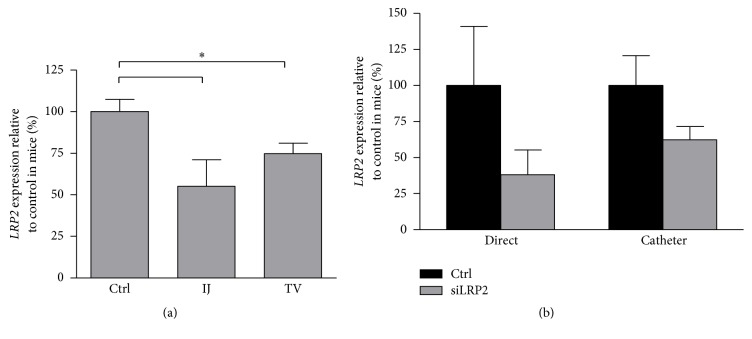
*LRP2* renal expression knockdown is not dependent upon administration site. (a) Significant knockdown was appreciated in mice receiving si*LRP2* through the internal jugular vein or the tail vein at 3 h (*n* = 15 control, *n* = 10 internal jugular (IJ), and *n* = 4 tail vein (TV)). Control mice include those receiving vehicle by internal jugular or tail vein injection. (b) For mice receiving si*LRP2* through the internal jugular vein, no difference was noted between those receiving direct IJ injections (*n* = 3 control and si*LRP2*) and those receiving siRNA through an IJ catheter (*n* = 7 control, and si*LRP2*). ^*∗*^*p* < 0.05 as compared to the control (Ctrl).

We considered that siRNA delivered through tail vein injections would enter hepatic circulation first and this might impact efficacy. Thus, we examined the difference in degree of mRNA expression reduction in mice given si*LRP2* through tail vein and internal jugular vein injections. Mice received a single dose of si*LRP2* or vehicle and were sacrificed 3 h after administration. Mice receiving internal jugular administration of siLRP2 had lower LRP2 expression (55.1 ± 16.0%) as compared to control mice (*p* = 0.019). No significant difference in knockdown was appreciated between mice receiving internal jugular injections and those receiving tail vein injections (*p* = 0.62). In mice receiving either tail vein or internal jugular injections of si*LRP2*, both had significantly lower expression of* LRP2* 3 h after administration as compared to control mice (Figure 4(a)).

Internal jugular injections were administered through direct injection via a cutdown procedure or through an implanted internal jugular catheter. We compared administration from an internal jugular vein cut down or through an internal jugular catheter (Figure 4(b)). Neither technique individually reached statistical significance as compared to control mice due to variability in control* LRP2* expression.* LRP2* expression did not significantly vary between techniques. Mice receiving a direct injection of siRNA into the internal jugular had 38.2 ± 17.0% of control expression and mice receiving siRNA through a catheter had 62.7 ± 9.2% expression of control mice (*p* = 0.20). Thus, the findings in Figure 4(a) were pooled from both internal jugular vein administration procedures to show significant* LRP2* knockdown as compared to control mice.

### 3.5. *In Vivo* siLRP2 Is Effective at Reducing* LRP2* mRNA Expression in Mouse Liver

**Figure 5:**
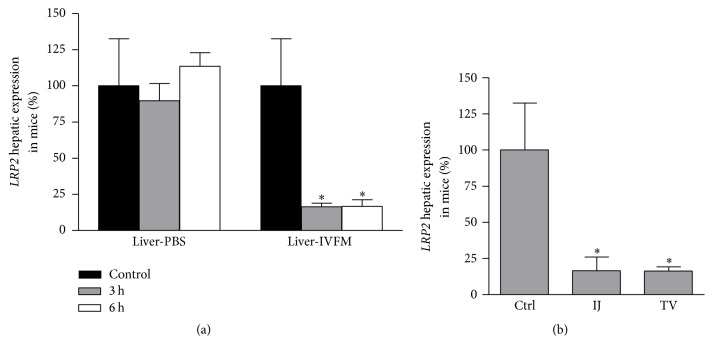
*In vivo* si*LRP2* is effective at reducing* LRP2* gene expression in mouse liver. (a) In mouse liver, si*LRP2* prepared in the carrier molecule Invivofectamine (IVFM) had lower measured* LRP2* expression than control mice or those receiving si*LRP2* in PBS (*n* = 5 control, *n* = 6 PBS carrier at 3 h, *n* = 5 IVFM carrier at 3 h, *n* = 3 of IVFM, and PBS at 6 h). (b) Mice receiving si*LRP2* by internal jugular (IJ) or tail vein (TV) injection both achieved reduced* LRP2* expression in liver. ^*∗*^*p* < 0.05 as compared to the control (Ctrl).

Although significant mRNA expression knockdown was appreciated in kidney tissue, the degree of knockdown was modest. Ambion's* in vivo* siRNA is optimized for hepatic expression reduction [32]. In the renal proximal tubule, siRNA dissolved in molecular grade PBS can be reabsorbed via endocytosis after it is filtered through the glomerulus [9]. In contrast, a carrier molecule is often required for hepatic uptake [32]. As a positive control for siRNA efficacy, we examined the degree of* LRP2* knockdown in liver tissue 3 h or 6 h after a single administration of si*LRP2* (Figure 5). siRNA was prepared in either PBS or the carrier molecule, Invivofectamine (IVFM). si*LRP2* in PBS carrier did not lead to a reduction in hepatic LRP2 expression as compared to control mice at either 3 h or 6 h (89.7 ± 12.0% expression at 3 h, *p* = 0.61; 113.6 ± 9.4% expression at 6 h, *p* = 0.74). In contrast, si*LRP2* in IVFM led to a significant reduction in expression down to 16.3 ± 2.6% as compared to control mouse expression (*p* = 0.0014). The expression reduction was still present 6 h after administration (reduction to 16.6 ± 4.6%, *p* = 0.011).

We tested whether administration site impacted hepatic* LRP2* knockdown at 3 h. Tail vein si*LRP2* administration with Invivofectamine reduced expression to 16.3 ± 3.0%  (*p* = 0.0016) and internal jugular administration reduced expression to 16.5 ± 9.5%  (*p* = 0.010) of control expression. No difference was observed between the administration sites (*p* = 0.94).

*LRP2* expression was also measured in lung tissue and whole blood at 3 h and 6 h after si*LRP2* administration. However, raw baseline expression CT values ranged from 31 to 35 and were undetectable in some specimens. No conclusions could be drawn regarding knockdown effect in these tissues.

### 3.6. Invivofectamine Does Not Improve Renal Efficacy of si*LRP2*

**Figure 6:**
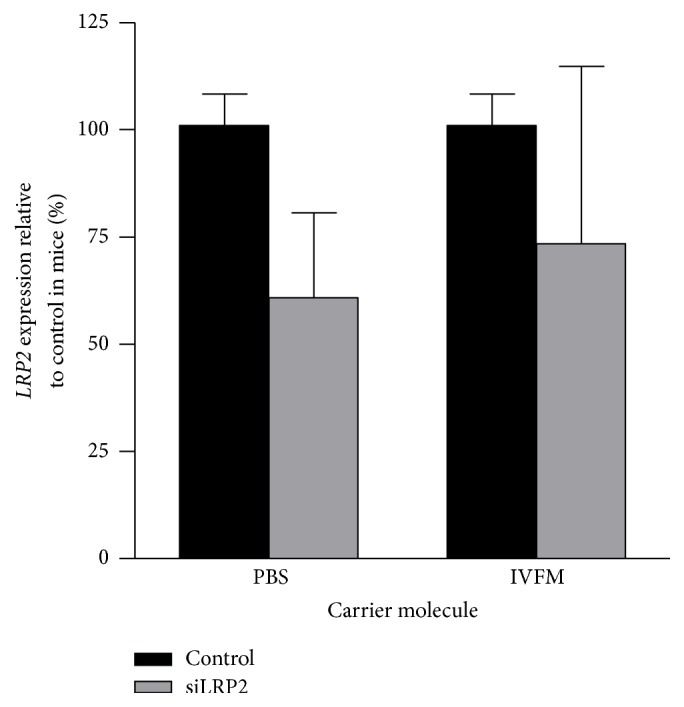
Invivofectamine does not improve renal efficacy of si*LRP2*. As compared to si*LRP2* prepared in PBS, preparing si*LRP2* in Invivofectamine did not significantly enhance mRNA knockdown (*n* = 3 to 4 per group). Due to the small number of mice used, no statistical difference was observed as compared to control mice.

Since si*LRP2* prepared in Invivofectamine led to a significant and impressive knockdown in hepatic tissue, we examined whether this carrier molecule could improve reduction of expression in the kidney. Mice were given si*LRP2* through the internal jugular vein and sacrificed 3 h later for expression measurements (Figure 6). siRNA was prepared in either PBS or Invivofectamine. Compared to control mice, si*LRP2* in Invivofectamine did not significantly reduce renal expression of* LRP2* (expression reduced to 73.5 ± 41.4% of control expression, *p* = 0.72). si*LRP2* in PBS reduced mRNA expression at a similar magnitude to previous experiments at 60.8 ± 20.0% expression of control (also nonsignificant with *p* = 0.11).

### 3.7. si*LRP2* Did Not Reduce Renal Megalin Protein Expression

**Figure 7:**
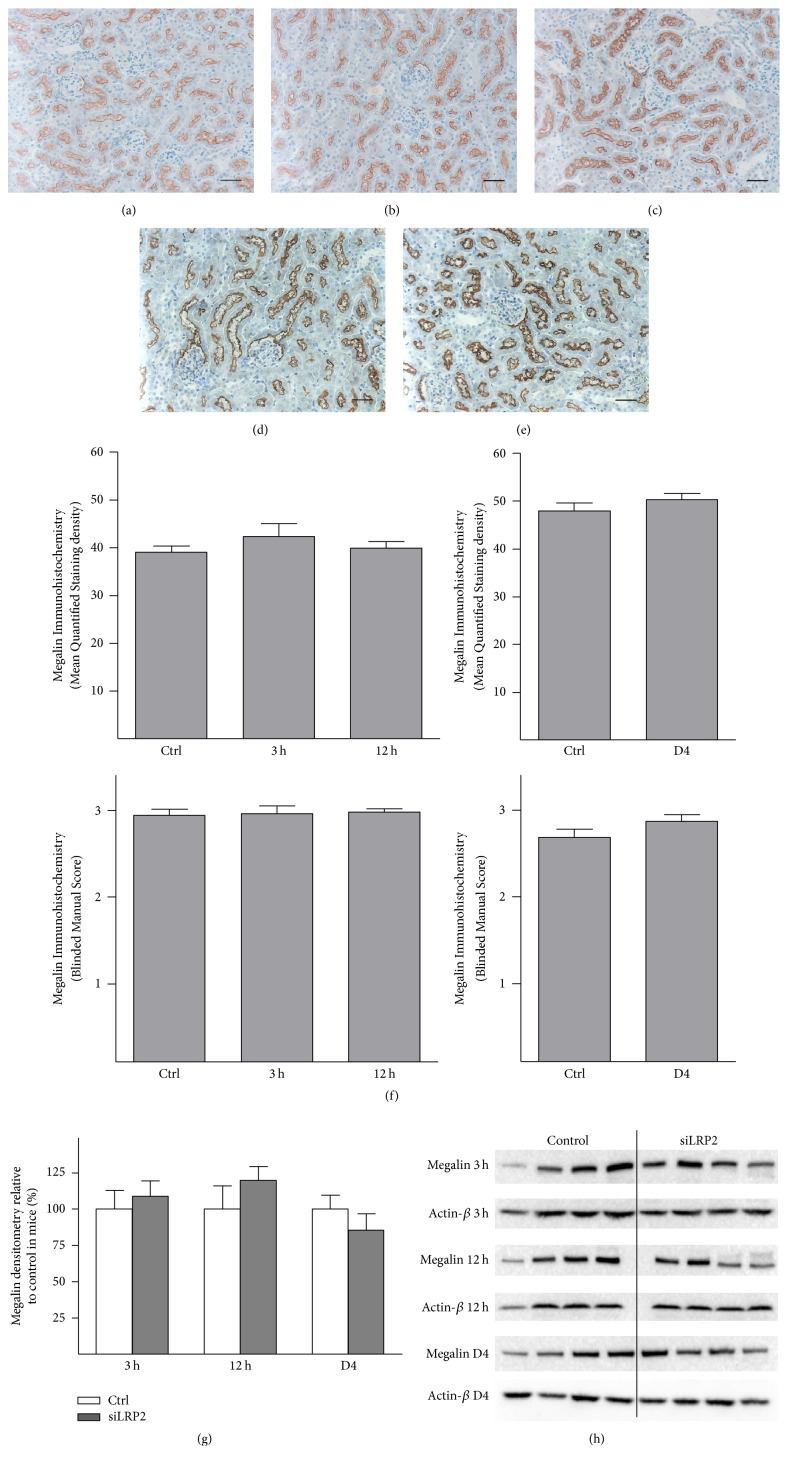
si*LRP2* did not reduce renal megalin protein expression 3 h or 12 h after a single dose. (a) Immunohistochemistry (IHC) of a control mouse. (b) IHC 3 h after administration of si*LRP2*. (c) IHC 12 h after administration of si*LRP2. *(d) IHC of a control mouse after 3.5 days of PBS vehicle. (e) IHC 3 h after a final seventh administration of si*LRP2*. (f) IHC staining density as quantified showed no significant difference; IHC staining density by visual scoring also revealed no significant difference. (g) Immunoblot densitometry of megalin in mice receiving si*LRP2*. (h) Immunoblots of megalin and actin for mice. *N* = 4 per group for all analyses. Control mice for the 3 h and 12 h time points were the same and sacrificed 3 h after vehicle administration (all three groups were sacrificed the same day). For IHC, 10 images were scored for each mouse. All images 20x, measurement bar is 50 *μ*m.

We studied the effect of si*LRP2* administration on renal cortical protein expression of 600 kDa megalin (Figure 7). Mice were given either vehicle or si*LRP2* in PBS through an IJ catheter and sacrificed at 3 h or 12 h after administration.* LRP2* expression was provided in Figure 3(c). We assessed protein expression by immunohistochemistry. By quantitative analysis with ImageJ, immunohistochemical staining for megalin revealed unchanged cortical expression in proximal tubules (*p* = 0.27 and 0.33 compared to control, resp.). Blinded microscopy supported this finding (*p* = 0.65 and 0.14 compared to control, resp.). Immunoblot of cortical kidney protein extract revealed unchanged protein expression at both time points.

We hypothesized that serial administration of si*LRP2* may be required to reduce megalin expression. si*LRP2* in PBS was administered every 12 hours via an IJ catheter for seven total doses. Mice were sacrificed 3 h after the seventh dose on day 4 (D4) and compared to mice receiving vehicle alone for 3.5 days. Megalin protein expression remained unchanged as measured by quantitated ImageJ IHC (*p* = 0.30), blinded microscopy (*p* = 0.22), and protein immunoblot with densitometry (*p* = 0.72).

## 4. Discussion

In this investigation, we sought to determine whether* in vivo* administration of siRNA would succeed in reducing expression of the constitutively expressed gene,* LRP2*, and its associated protein, megalin. This endeavor builds upon several prior investigations [10–14]. These investigations have succeeded in preventing upregulation of gene expression in response to a stimulus or disease state. In contrast, we were able to reduce* baseline* mRNA expression, using 6-fold higher doses of siRNA. Reduction of* LRP2* expression was attained in mice, but a concomitant protein expression decrease was not observed, even after serial administration. A number of factors may contribute to this finding, including (1) the high proportion of membrane-bound protein as compared to cytoplasmic protein, (2) the modest degree of mRNA reduction, (3) the rapid rebound and increase in mRNA expression following siRNA delivery, and (4) the relatively long half-life of the protein (4.8 h) [25]. These results support the theoretical use of siRNA to transiently reduce baseline expression of transcripts in the kidney; however, the results also suggest that the practical application of this technique will prove difficult.

Our results build upon the existing body of literature. We have shown that* LRP2* rapidly rebounds to a level of mRNA expression which is significantly above baseline expression. This finding has been previously reported in mice for other genes. For example, RNAi targeting the secretory hepatitis B virus surface antigen gene led to an initial gene knockdown followed by an expression rebound [33]. The authors identified upregulation of the meri-1 (mouse enhanced RNAi) and adar-1 (adenosine deaminase acting on RNA) genes as a potential mechanistic explanation. One key difference is that the mRNA rebound effect occurred after 4–7 days of knockdown. In contrast, we identified rebound and upregulation as early as 12 h following administration.

Several limitations affect the generalizability of our results. Foremost amongst these limitations was the inability to reduce protein expression. As we discuss above, the membrane to cytoplasm ratio of the protein, modest mRNA knockdown effect, rapid rebound effect, and prolonged half-life all contributed to siRNA inefficacy. However, several unique features of megalin and* LRP2* may additionally conspire to impair gene and protein knockdown. First, megalin has been shown to mediate uptake of siRNA in proximal tubule cells [34]. While this may appear counterintuitive to knock down the very protein responsible uptake, in this scenario, it is unlikely to have had a remarkable effect since (1) megalin's uptake capacity is high, and (2) protein expression reduction was never achieved. A second factor that may have affected efficacy is the rapid and repetitive recycling of the protein [25]. Megalin recycles from the cytoplasm to the cell membrane every 1.2 minutes. This factor should not have changed megalin's degradation half-life of 4.8 h, but it might affect gene expression regulation. Megalin mRNA expression has been shown to be regulated by its compartmental protein expression [35]. Overexpression of both the membrane-bound megalin COOH-terminal fragment (MCTF) and the soluble megalin intracellular domain (MICD) lead to significantly lower levels of megalin mRNA. Of note, the antibody used in our study maps to the carboxy-terminal of megalin. MCTF is cleaved by gamma-secretase into MICD and inhibition of gamma-secretase has been showed to restore megalin mRNA expression in MCTF overexpressed proximal tubular cells. Since blocking formation of MICD leads to increased megalin gene and protein expression, the opposite may hold true. Thus, if soluble intracellular megalin levels are decreased by siRNA, it follows that we might expect a significant counterregulatory increase in gene and protein expression.

Ultimately, we were not able to reach our goal of using si*LRP2* as a therapeutic alternative to cilastatin or RAP. Both cilastatin and RAP have been used to block or decrease expression of megalin in the proximal tubule, preventing reabsorption of nephrotoxic compounds [21, 22]. Several alternative approaches can be considered in future experimentation. Nanocarrier molecules [15, 16] have been used to enhance renal delivery and efficacy of siRNA. These molecules have been used to prevent upregulation of expression in response to injury or disease but are not yet widely available. Their use holds potential to improve the degree of mRNA expression reduction. However, increased degree of knockdown does not overcome other factors like the extended protein half-life, rapid recycling to and from the membrane, rebound mRNA expression, and complex regulation of expression by the intracellular protein levels.

Approaches that might counterbalance the half-life and rebound expression include a continuous infusion of siRNA or more frequent dosing. The 12-hour time point for serial administration was chosen because of volume administration limitations, feasibility, and cost. The mRNA rebound phenomenon observed may have impaired the ability to achieve protein knockdown. While it is possible that a continuous infusion or more frequent dosing of siRNA would succeed in reducing megalin protein expression, cilastatin and RAP are better alternatives to focus on to reduce nephrotoxicity. Since both cilastatin and RAP can be administered once daily, si*LRP2* is not an efficient or cost-effective means of preventing nephrotoxicity.

## 5. Conclusions

In conclusion, we demonstrated that si*LRP2* administration was sufficient to modestly reduce renal mRNA expression in mice. This mRNA reduction did not lead to a reduction in megalin protein expression. A number of very strong publications have illustrated the use of siRNA to mitigate renal injury. Publication bias of positive results remains relevant in biomedical research. Although this investigation was encumbered by a number of limitations, we believe the data presented will assist other investigators in optimizing future RNAi experimentation with the kidney. The RNAi therapeutic approach may remain more suited to prevention of upregulation than reduction of baseline mRNA expression.

## Figures and Tables

**Table 1:** Primer sequences.

Molecule	Sequence (5′-3′)
*LRP2* F primer	CCT TGC CAA ACC CTC TGA AAA T
*LRP2* R primer	CAC AAG GTT TGC GGT GTC TTT A
*18S* F primer	GTT GGT GGA GCG ATT TGT CT
*18S* R primer	GAA CGC CAC TTG TCC CTC TAT
